# GAIT SLOWING ON TRANSITION TO UNEVEN SURFACES: DOPAMINERGIC NEUROTRANSMISSION AND PERIPHERAL SYSTEM RISK FACTORS

**DOI:** 10.1093/geroni/igae098.1058

**Published:** 2024-12-31

**Authors:** Caterina Rosano, Andrea Rosso, Anne Newman, Stephen Cummings, Stephanie Studenski, Nicholaas Bohnen, Lana Chahine

**Affiliations:** University of Pittsburgh, Pittsburgh, Pennsylvania, United States; University of Pittsburgh, Pittsburgh, Pennsylvania, United States; University of Pittsburgh, University of Pittsburgh, Pennsylvania, United States; University of California San Francisco, San Francisco, Mississippi, United States; University of Pittsburgh, Pittsburgh, Pennsylvania, United States; University of Michigan, Ann Arbor, Michigan, United States; University of Pittsburgh, Pittsburgh, Pennsylvania, United States

## Abstract

Identifying mechanisms that compensate for slow gait speed in older adults is crucial. Dopaminergic neurotransmission curbs deleterious associations of cerebrovascular disease with gait, but whether it compensates for peripheral systemic risk factors (PSRF) for gait slowing has not been studied. In this cross-sectional study of community-dwelling older adults, we examined the relationship between nigrostriatal dopaminergic terminal integrity and gait speed in individuals with and without ≥1 PSRF for gait slowing: obesity, joint pain, or reduced muscle strength. The primary outcome was percent change in gait speed (%CGS) on transition from even to uneven surface. Participants underwent dopaminergic imaging with dihydrotetrabenazine [11C]DTBZ positron emission tomography. Among 199 individuals, (mean (SD) age 75.0 (4.6) years; 62.3% female; 91.0% White) 131 (65.8%) had ≥1 PSRF. Relationship between posterior putamen [11C]DTBZ binding and %CGS was modified by PSRF; in those with ≥1 PSRF (but not in those with no PSRF), posterior putamen [11C]DTBZ binding was associated with %CGS (β=0.238, p=0.01) independent of potential confounders. Striatal dopaminergic neurotransmission may offer resilience to effects of PSRF on gait slowing.

